# Attenuated rightward hemispheric asymmetry in ADHD: structural MRI evidence from a normalized asymmetry index and its association with cognitive performance

**DOI:** 10.3389/fnins.2026.1764242

**Published:** 2026-03-12

**Authors:** Metin Çınaroğlu, Eda Yılmazer, Selami Varol Ülker, Sultan Tarlacı

**Affiliations:** 1 Department of Psychology, Faculty of Administrative and Social Science, Istanbul Nişantaşı University, Istanbul, Türkiye; 2 Department of Psychology, Faculty of Social Science, Beykoz University, Istanbul, Türkiye; 3 Department of Psychology, Faculty of Humanities and Social Science, Üsküdar University, Istanbul, Türkiye; 4 Medical School, Üsküdar University, Istanbul, Türkiye

**Keywords:** ADHD, cerebellum, cortical thickness, hemispheric asymmetry, MOXO-CPT, neurodevelopment, structural MRI

## Abstract

**Background:**

Altered hemispheric asymmetry has been proposed as a potential neurodevelopmental feature of Attention-Deficit/Hyperactivity Disorder (ADHD). However, findings remain inconsistent, and the functional relevance of structural asymmetry patterns is not well established. This study examines volumetric and cortical-thickness asymmetries across cortical and subcortical regions in children and adolescents with ADHD compared to typically developing controls and evaluates their association with objective cognitive performance.

**Methods:**

Forty participants with ADHD and 30 age- and sex-matched controls underwent high-resolution T1-weighted MRI. Bilateral regional volumes and cortical thickness were quantified using the volBrain pipeline, and asymmetry indices (AI = [R–L]/[(R + L)/2]) were computed for lobar and subcortical structures. Group differences were assessed using independent *t*-tests. Within the ADHD group, associations between asymmetry indices and MOXO-d-CPT performance (Attention, Timing, Impulsivity, Hyperactivity) were examined using Pearson correlations with correction for multiple comparisons.

**Results:**

ADHD participants showed significantly reduced rightward asymmetry in frontal lobe volume, cerebellar hemispheres, caudate, putamen, and amygdala (ps < 0.05). Cortical-thickness asymmetry was also diminished in the frontal and parietal lobes and the anterior cingulate cortex. Temporal and occipital asymmetries were preserved. Within the ADHD group, greater rightward frontal and ACC thickness asymmetry correlated with better attention performance (*r* = 0.45 and 0.40), rightward parietal asymmetry associated with more accurate timing (*r* = 0.38), reduced rightward IFG asymmetry related to greater impulsivity (*r* = −0.42), and amygdala asymmetry correlated with lower hyperactivity (*r* = 0.36).

**Conclusion:**

Children with ADHD exhibit a consistent attenuation of typical right-hemisphere dominance across frontal, striatal, cerebellar, and limbic systems. These altered asymmetry patterns are meaningfully associated with attentional control, timing accuracy, impulsivity, and hyperactivity, suggesting that hemispheric imbalance may serve as a structural may represent a neurodevelopmental characteristic associated with ADHD. Findings support models emphasizing right-hemisphere developmental lag and highlight hemispheric asymmetry as a clinically relevant dimension of ADHD neurobiology.

## 1 Introduction

ADHD is a common neurodevelopmental disorder of childhood, characterized by developmentally inappropriate levels of inattention, hyperactivity, and impulsivity (McClain et al., 2017). It affects an estimated 5–7% of children worldwide, with symptoms often persisting into adolescence and adulthood (Di Lorenzo et al., 2021). Clinically, ADHD is highly heterogeneous – individuals vary in symptom presentation (predominantly inattentive, hyperactive/impulsive, or combined types), severity, and comorbid conditions (Luo et al., 2019). This heterogeneity suggests that multiple neurobiological pathways underlie ADHD, making it critical to examine brain structure and organization for biomarkers and phenotypic clues (Bu et al., 2025; Halperin et al., 2016).

Neuroimaging studies indicate that children with ADHD have subtle but widespread alterations in brain structure (Kangarani-Farahani et al., 2022; Yu et al., 2023). Total cerebral volume is on average slightly smaller (roughly 3–5%) in ADHD youth compared to peers (Li et al., 2022). More specifically, volumetric reductions are most consistently reported in brain regions subserving executive function and motor control, including the frontal lobes, basal ganglia, and cerebellum (Leisman and Melillo, 2022). For example, a meta-analysis by Valera et al. (2007) found the largest case–control differences in ADHD were reduced right hemisphere brain volume (right cerebral hemisphere and right caudate nucleus) as well as smaller cerebellar volumes. These findings align with the theory that ADHD involves atypical development of fronto-striatal and fronto-cerebellar networks that support attention and inhibitory control (Durston et al., 2011). Indeed, prior MRI studies have repeatedly observed that children with ADHD have lower frontal lobe volumes—particularly in the right prefrontal cortex (Dirlikov et al., 2015)—consistent with either delayed cortical maturation or persistent under-development in these regions (Vaidya and Stollstorff, 2008). Likewise, the basal ganglia (especially the caudate and putamen) tend to be smaller in ADHD (Leisman and Melillo, 2022; Wellington et al., 2006), and these differences have been linked to symptom severity [e.g., smaller caudate volumes correlating with greater hyperactivity, (Qiu et al., 2009)]. The cerebellum, which is involved in motor timing and higher-order cognition, is also reduced in volume by ~4% in ADHD on average (Mahone, 2011). Together, such volumetric findings suggest that the neural circuits for executive function are structurally altered in ADHD (Albajara Sáenz et al., 2019). However, traditional case–control volumetric comparisons have yielded mixed or inconsistent results, partly due to high inter-individual variability (Rubia et al., 2021). This has prompted interest in *hemispheric asymmetry* measures as an alternative approach to characterizing neurodevelopmental differences in ADHD (He et al., 2022).

Hemispheric asymmetry is a fundamental principle of brain organization: in typical development, each hemisphere shows slight differences in size and cortical thickness that reflect specialization [for instance, language regions are larger in the left hemisphere, whereas attentional and visuo-spatial networks are often right-lateralized, (Shaw et al., 2009; Ocklenburg et al., 2024; Wang et al., 2023)]. Aberrant lateralization has been implicated in a number of neurodevelopmental disorders (Bradshaw and Sheppard, 2000), and ADHD is hypothesized to involve atypical right–left brain development (Friedman and Rapoport, 2015; Stefanatos and Wasserstein, 2001). Notably, many cognitive operations impaired in ADHD—such as sustained attention, response inhibition, and temporal processing—are functions generally associated with right-hemisphere networks in typically developing children (Amiri et al., 2024). This has led to models of ADHD as a “right hemisphere dysfunction” syndrome, supported by functional MRI findings of reduced activation in right frontal regions during inhibitory control tasks in ADHD (Baytunca et al., 2021; Boyraz et al., 2021). Structural MRI studies have also observed anomalous asymmetries in ADHD (Postema et al., 2021). For example, some reports suggest that certain structures that are normally larger in the right hemisphere show *reduced* rightward asymmetry or even a leftward bias in youth with ADHD (Tomasi and Volkow, 2025). A recent analysis found a reversal of the typical putamen asymmetry in ADHD (i.e., healthy children showed a slight right > left putamen, whereas ADHD children showed the opposite, Chen et al., 2021). Similarly, aberrant asymmetries have been noted in the caudate, amygdala, and thalamus of adolescents with ADHD (Baboli et al., 2022; Chen et al., 2023). These findings, while varied, converge on the notion that examining *within-person* right–left differences may reveal ADHD-related neuroanatomical deviations that are less apparent on absolute volume measures. In fact, using asymmetry indices (AIs) allows each individual to serve as their own control for overall brain size, potentially enhancing sensitivity to group differences (Williams et al., 2022). By studying whether the normal hemispheric dominance in key regions (e.g., right > left frontal volume) is attenuated in ADHD, we can examine hemispheric lateralization as a neurodevelopmental characteristic of ADHD.

## 2 Method

### 2.1 Study design

In the present study, we investigated hemispheric asymmetries in brain structure—specifically regional volumes and cortical thickness—in children and adolescents with ADHD. We employed an automated MRI analysis pipeline (volBrain) to obtain volumetric and cortical thickness measurements of each hemisphere’s frontal, parietal, temporal, occipital, cerebellar, and subcortical regions. The volBrain system provides reliable segmentation-based volumetry and asymmetry estimates referenced to age- and sex-matched norms, making it well suited for developmental samples.

Hemispheric asymmetry was quantified using a mean-normalized laterality formulation (AI = [R–L]/[(R+L)/2]) for each region of interest. This approach has been recommended in large-scale neuroimaging analyses of structural asymmetry (e.g., Kong et al., 2022) and in methodological evaluations comparing alternative asymmetry metrics (Williams et al., 2022). By normalizing hemispheric differences to the bilateral mean, this formulation reduces scaling bias related to inter-individual variability in overall regional volume and enhances interpretability in developmental populations.

In addition, recognizing that structural differences may have functional correlates, we examined whether the degree of hemispheric asymmetry was associated with cognitive performance within the ADHD group. To this end, we administered the MOXO-digital Continuous Performance Test (MOXO-d-CPT), a computerized attention task that provides standardized indices of Attention, Timing, Impulsivity, and Hyperactivity. By correlating regional asymmetry measures with MOXO subscale scores, we aimed to link neuroanatomical lateralization patterns with clinically relevant cognitive dimensions of ADHD.

### 2.2 Study aims and hypotheses

The primary aim of the present study was to examine hemispheric asymmetry patterns in ADHD using a mean-normalized laterality formulation. Rather than proposing a fundamentally new metric, we employed an explicit mean-normalized expression of the traditional laterality index to enhance interpretability and scale transparency in a developmental sample. While prior neuroimaging studies have reported volumetric alterations in ADHD, findings have been inconsistent, in part due to reliance on absolute left–right comparisons that do not adequately account for inter-individual variability in brain size. To address this limitation, we employed a normalized asymmetry index (AI = [R–L]/[(R+L)/2]), allowing each participant to serve as their own internal control. This approach enhances sensitivity to lateralization differences independent of overall regional volume and is particularly suitable for developmental samples.

Using this normalized asymmetry framework, our first aim was to determine whether children and adolescents with ADHD exhibit attenuated rightward hemispheric asymmetry across cortical and subcortical systems implicated in attention and executive control. We focused on frontal, parietal, temporal, and occipital lobes, as well as cerebellar hemispheres and key subcortical nuclei (caudate, putamen, thalamus, amygdala, hippocampus). Based on neurodevelopmental models emphasizing right-hemisphere involvement in attention and inhibitory control, we hypothesized that ADHD would be characterized by a blunting or reversal of typical rightward asymmetry in fronto-striatal, fronto-cerebellar, and limbic regions.

#### 2.2.1 Hemispheric volume asymmetry

We hypothesized that youth with ADHD would exhibit reduced rightward asymmetry in regional brain volumes compared to healthy controls. Specifically, we expected diminished right-greater-than-left volume in the frontal lobe, basal ganglia (caudate and putamen), and cerebellar hemispheres, reflecting atypical development of right-lateralized executive networks. The occipital lobe was included as a reference region, and we anticipated no group difference in occipital asymmetry given its typically symmetric organization.

#### 2.2.2 Hemispheric cortical thickness asymmetry

We further hypothesized that a similar attenuation pattern would be observed for cortical thickness. In particular, we expected reduced rightward asymmetry (or a relative leftward shift) in frontal and parietal cortical thickness in the ADHD group. Given evidence that frontal regions typically show slight rightward thickness advantages during development, an absence or reversal of this pattern would be consistent with a right-hemisphere developmental lag model. We also examined asymmetry in specific executive-control regions, including the anterior cingulate cortex (ACC) and inferior frontal gyrus (IFG), predicting reduced right-dominance in these areas in ADHD.

#### 2.2.3 Behavioral correlates of asymmetry

Our second major aim was to evaluate the functional significance of structural asymmetry. We hypothesized that individual differences in hemispheric asymmetry would be meaningfully associated with cognitive performance in the ADHD group. Specifically, a more pronounced rightward asymmetry (i.e., a more typical lateralization profile) in fronto-cortical regions was expected to predict better attentional and inhibitory performance on the MOXO-d-CPT. Conversely, reduced rightward asymmetry or a leftward bias was expected to relate to poorer performance, including greater inattention and impulsivity.

For example, we predicted that lower frontal lobe AI values (reflecting reduced rightward dominance) would be associated with lower MOXO Attention scores. Similarly, reduced rightward asymmetry in the IFG and ACC—regions central to impulse control and sustained attention—was expected to correlate with higher Impulsivity scores and lower Attention scores, respectively. Finally, we explored whether asymmetries in subcortical structures involved in arousal and motor regulation (e.g., amygdala and thalamus) would relate to Hyperactivity scores.

### 2.3 Participants

The study sample consisted of 40 children and adolescents diagnosed with ADHD (per DSM-5 criteria) and 30 age- and sex-matched healthy control participants. ADHD participants were recruited from a university-affiliated child and adolescent psychiatry clinic, whereas control participants were recruited from the community to closely match the patient group’s demographic characteristics. The groups did not differ significantly in mean age (approximately 12.5 vs. 12.8 years) or sex distribution (~70% male in each group). All participants were right-handed and had no known neurological or psychiatric comorbidities beyond ADHD. Additional exclusion criteria included any history of significant head trauma, intellectual disability, or contraindications to MRI (e.g., metal implants or claustrophobia). ADHD diagnoses were established by a licensed psychiatrist using DSM-5 diagnostic criteria. Written informed consent was obtained from each participant’s parent or guardian (with assent from minors), and the study protocol was approved by the university hospital’s institutional ethics committee. At the time of MRI acquisition, 29 participants with ADHD were medication-naïve, while 11 were receiving pharmacological treatment. Specific medication types and dosages were not systematically recorded. No formal washout period was implemented prior to scanning.

### 2.4 MRI acquisition

Neuroimaging was performed using a 1.5 Tesla MRI scanner with a high-resolution T1-weighted 3D magnetization-prepared rapid acquisition gradient-echo (MPRAGE) sequence. Example MRI parameters for the MPRAGE acquisition were: repetition time (TR) = 2,800 ms, echo time (TE) = 4.0 ms, flip angle = 8°, field of view = 240 mm, acquisition matrix = 256 × 256, with 135 contiguous slices yielding an isotropic voxel size of approximately 0.94 mm × 0.94 mm × 1.2 mm. This protocol provided whole-brain coverage of anatomical images for subsequent analysis.

#### 2.4.1 Image processing and asymmetry analysis

Structural MRI data were processed using volBrain (volbrain.upv.es), an automated neuroimaging platform for segmentation-based volumetric and cortical thickness quantification. For each participant, volBrain generated volumetric estimates of total and lobar brain regions as well as cortical thickness measurements across the cortex.

Bilateral volume measures were extracted for each cerebral lobe (frontal, parietal, temporal, occipital), cerebellar hemispheres, and key subcortical nuclei (caudate, putamen, thalamus, amygdala, hippocampus). These region-of-interest measures formed the basis for hemispheric asymmetry analyses.

For each homologous left–right pair, a hemispheric Asymmetry Index (AI) was computed using the mean-normalized formula:


$$\mathrm{AI}=\frac{R-L}{(R+L)/2}$$

where *R* and *L* denote the right and left regional measurements, respectively. This formulation expresses hemispheric difference relative to the bilateral mean, yielding a symmetric and scale-independent metric of lateralization (Wyciszkiewicz and Pawlak, 2014). Positive values may indicate rightward asymmetry (R > L), negative values may indicate leftward asymmetry (L > R), and values near zero reflect structural symmetry. This formulation is algebraically equivalent to 2 × LI, where LI = (R−L)/(R+L). Because correlation coefficients and classification metrics are invariant under linear transformation, this formulation does not alter statistical inference relative to the traditional laterality index, but enhances interpretative transparency by explicitly normalizing hemispheric difference to the bilateral mean.

Asymmetry indices were calculated for all volumetric measures (lobar, subcortical, cerebellar). For cortical thickness, analogous AIs were computed at the lobar level using mean hemispheric thickness values. This procedure yielded a standardized lateralization metric for each structural region of interest.

All structural images underwent quality control prior to analysis. Raw MRI scans were visually inspected for excessive motion artifacts, incomplete brain coverage, and major imaging distortions. Following automated segmentation with volBrain, segmentation outputs were reviewed for anatomical plausibility and absence of gross misclassification or processing failures. Scans that did not meet quality criteria were excluded from further analysis. Only datasets deemed suitable for reliable volumetric and cortical thickness estimation were included in asymmetry computations.

In addition to visual and segmentation quality control, we examined the volBrain “scale factor” parameter, which reflects the global affine scaling applied during normalization of each T1-weighted image to standard template space. The scale factor values did not differ between ADHD and control groups (*p* = 0.991), indicating comparable global scaling and supporting the absence of systematic normalization bias between groups. Detailed information regarding scale factor interpretation and group comparisons is provided in Supplementary Methods S2.

### 2.5 Behavioral assessment

All statistical analyses were conducted using IBM SPSS Statistics version 26 and JASP version 0.9. Group comparisons of asymmetry indices were performed using general linear models with age and sex entered as covariates. This approach was applied separately to each regional AI (volumetric and cortical thickness measures). Within the ADHD group, associations between asymmetry indices and MOXO-d-CPT scores were examined using partial Pearson correlations controlling for age and sex. All participants completed the MOXO-d-CPT, a computerized continuous performance test designed to objectively assess attention and impulse control. The MOXO-d-CPT (NeuroTech Solutions) is a 15-min, game-like test administered on a computer, suitable for ages 6 and up. During the test, participants are required to respond to certain target stimuli by pressing a key, while refraining from responding to non-target stimuli, all presented amid various visual and auditory distractors. The task is divided into successive stages that introduce increasing levels of distraction, challenging the participant’s sustained attention and inhibitory control. The MOXO-d-CPT provides four outcome indices corresponding to core attention-related domains: Attention, Timing, Impulsivity, and Hyperactivity. The Attention score primarily may reflect attentional performance (e.g., fewer omission errors may indicate better attention), while the Impulsivity score may reflect the number of commission errors (responses to non-targets). The Timing score indexes the accuracy of response timing (responding neither too early nor too late), and the Hyperactivity score captures inappropriate or excessive responses (such as impulsive key presses beyond specific commission errors). Each MOXO index is standardized for the participant’s age group and sex, with higher scores indicating better performance (i.e., fewer attentional lapses, more consistent timing, and fewer impulsive or hyperactive responses). The test was administered in a quiet room according to standard instructions, and MOXO performance scores for the four subscales were recorded for analysis. Detailed scoring procedures for each MOXO-d-CPT subscale (Berger et al., 2013) are provided in Supplementary Methods S1.

### 2.6 Statistical analysis

All statistical analyses were conducted using IBM SPSS Statistics version 26 and JASP version 0.9. Group comparisons of asymmetry indices (AI) between the ADHD and control groups were performed using independent-samples *t*-tests (two-tailed), with age and sex included as covariates in general linear models where appropriate. This procedure was applied separately to each regional AI (volumetric and cortical thickness measures).

Prior to analysis, the assumptions underlying independent-samples t-tests were evaluated. Normality of AI distributions within each group was assessed using the Shapiro–Wilk test, which indicated no significant deviations from normality for any region (all *p* > 0.05). Homogeneity of variances was evaluated using Levene’s test and was satisfied for the majority of regions. To ensure robustness against potential variance inequality, all group comparisons were additionally conducted using Welch’s *t*-test. Results obtained using Welch’s procedure were fully consistent with those from the standard independent-samples t-tests, with all significant findings remaining statistically significant. For clarity and comparability with prior literature, results from the standard independent-samples t-tests are reported.

Within the ADHD group, associations between regional asymmetry indices and MOXO-d-CPT performance (Attention, Timing, Impulsivity, Hyperactivity) were examined using partial Pearson correlation analyses controlling for age and sex. An alpha level of 0.05 was used to determine statistical significance. Given the number of brain regions and behavioral measures examined, Bonferroni correction was applied to adjust for multiple comparisons, thereby reducing the risk of Type I error. All reported *p*-values reflect corrected thresholds where applicable. Effect sizes (Cohen’s d for group comparisons and Pearson’s r for correlations) were calculated to facilitate interpretation of the magnitude of observed effects.

## 3 Results

### 3.1 Participant characteristics

A total of 40 children and adolescents with ADHD and 30 healthy controls were included in the analyses. As shown in Table 1, groups did not differ significantly in age, sex distribution, handedness, or total intracranial volume (all *p* > 0.20). No significant main effects of sex or Group × Sex interactions were observed for any asymmetry index (all *p* > 0.10); therefore, subsequent analyses were conducted on the combined sample within each group.

**Table 1:** Demographic and clinical characteristics of the ADHD (DEHB) and healthy control groups.

Variable	ADHD (*n* = 40)	Controls (*n* = 30)	*t* / χ^2^	*p*
Age (years)	12.5 ± 2.1	12.8 ± 2.3	0.59	0.56
Sex (male/female)	28/12	20/10	0.09	0.77
Handedness (right/left)	37/3	28/2	0.01	0.94
Total intracranial volume (cm^3^)	1,415 ± 125	1,428 ± 119	0.39	0.70
MOXO attention (mean ± SD)	32.8 ± 6.4	38.2 ± 5.9	3.61	0.001
MOXO timing (mean ± SD)	29.4 ± 5.8	35.1 ± 6.2	4.02	< 0.001
MOXO impulsivity (mean ± SD)	24.3 ± 7.6	28.1 ± 6.9	2.00	0.049
MOXO hyperactivity (mean ± SD)	26.9 ± 8.0	32.7 ± 7.4	3.02	0.004
Full-scale IQ	98.5 ± 11.8	101.3 ± 10.7	0.98	0.33

### 3.2 Volumetric hemispheric asymmetries

**Figure 1:**
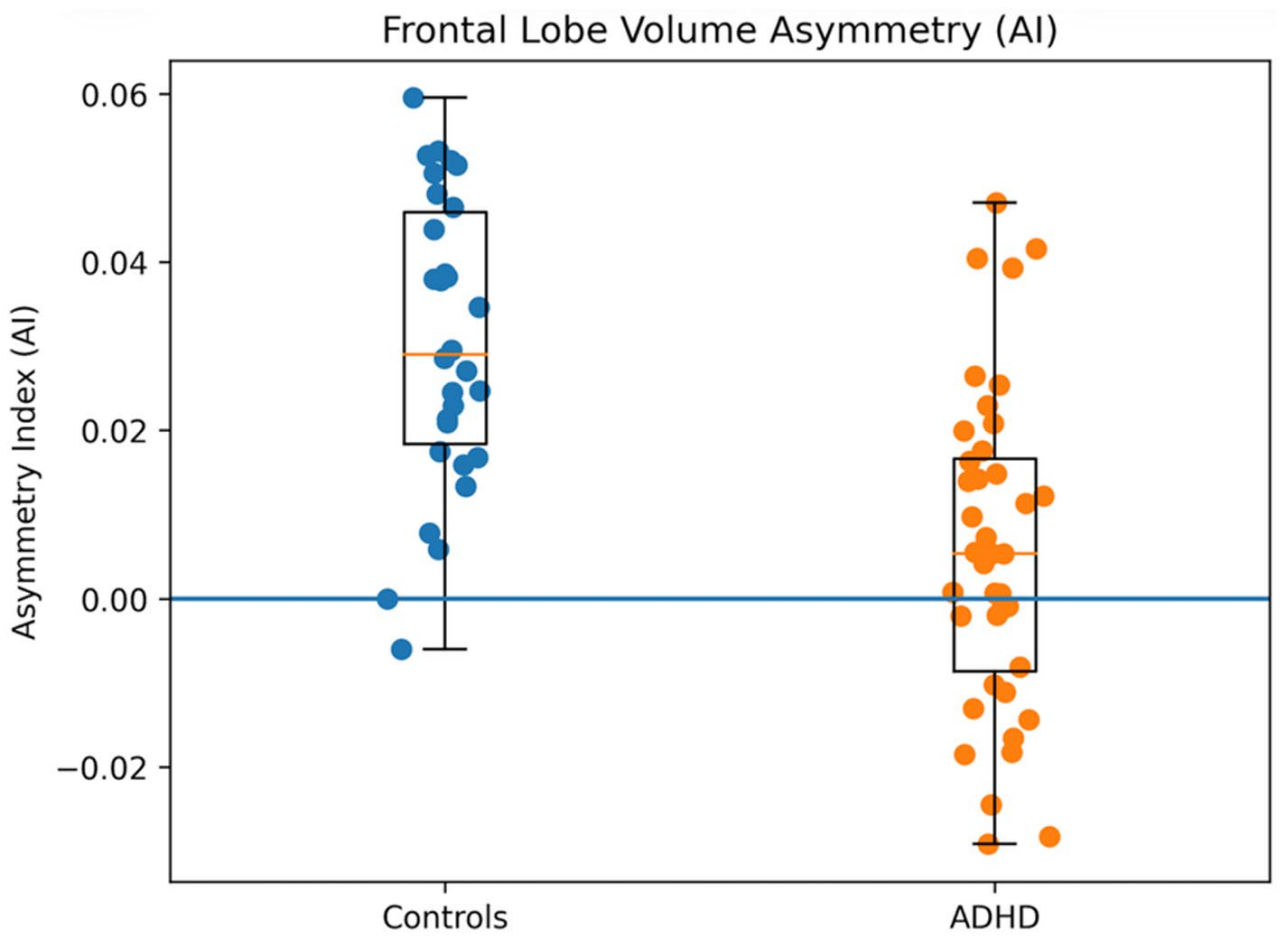
Frontal lobe volume asymmetry (AI) in ADHD and control groups.

Group comparisons of volumetric asymmetry indices (AI) revealed several significant differences between the ADHD and control groups (Table 2). In the frontal lobe, the volume AI was significantly lower in the ADHD group than in controls, *t*(66) ≈ 3.10, *p* = 0.003, *d* ≈ 0.75. On average, controls showed a rightward asymmetry in frontal lobe volume (AI > 0, indicating larger right frontal volume), whereas the ADHD group had a greatly reduced or absent rightward asymmetry (AI near 0). In fact, the ADHD group’s mean frontal volume AI was close to zero (M ≈ +0.01, SD ≈ 0.02), compared to a moderate right-dominant asymmetry in controls (M ≈ +0.03, SD ≈ 0.02). This finding suggests that the typical right-greater-than-left frontal volume pattern is attenuated in ADHD. Figure 1 illustrates this group difference in frontal lobe AI, with ADHD participants showing a significantly more symmetric frontal volume relative to healthy peers.

**Table 2:** Volumetric asymmetry indices (AI) by region in ADHD (DEHB) and healthy control groups.

Brain region	AI mean ± SD (ADHD, *n* = 40)	AI mean ± SD (controls, *n* = 30)	*t*(66)	*p*	Cohen’s *d*	Direction of asymmetry (HC vs. ADHD)
Frontal lobe volume	+0.010 ± 0.020	+0.031 ± 0.021	3.10	0.003	0.75	Rightward ↓ in ADHD
Parietal lobe volume	+0.006 ± 0.017	+0.008 ± 0.018	0.42	0.67	0.10	ns
Temporal lobe volume	+0.004 ± 0.015	+0.006 ± 0.016	0.47	0.64	0.12	ns
Occipital lobe volume	0.000 ± 0.012	0.000 ± 0.011	0.01	0.99	0.00	Symmetric (control check)
Cerebellar hemispheres	−0.003 ± 0.019	+0.012 ± 0.018	2.45	0.017	0.60	Leftward shift in ADHD
Caudate nucleus	+0.002 ± 0.014	+0.018 ± 0.016	2.90	0.005	0.70	Rightward ↓ in ADHD
Putamen	+0.004 ± 0.015	+0.020 ± 0.018	2.60	0.011	0.63	Rightward ↓ in ADHD
Amygdala	−0.007 ± 0.013	+0.006 ± 0.014	2.20	0.031	0.52	Reversed (Left > Right) in ADHD
Thalamus	+0.001 ± 0.012	+0.008 ± 0.013	1.83	0.07	0.45	Trend (Rightward ↓)
Hippocampus	+0.004 ± 0.015	+0.007 ± 0.015	0.77	0.44	0.19	ns

AI, Asymmetry Index; HAI, Hemispheric Asymmetry Index; ACC, Anterior Cingulate Cortex; IFG, Inferior Frontal Gyrus; MOXO-d-CPT, MOXO-digital Continuous Performance Test; SD, Standard Deviation; ns, non-significant.

Volumetric AI differences were observed in other regions as well. The cerebellar hemispheres showed a significant group difference in volume asymmetry, *t*(66) ≈ 2.45, *p* = 0.017. Controls exhibited a mild rightward bias in cerebellar volume (right > left), whereas the ADHD group showed a near symmetric or slightly leftward cerebellar volume (AI slightly negative). Similarly, the ADHD group had striatal asymmetries that differed from controls (Table 2 and Figure 1). The volume AI for the caudate nucleus was significantly lower in ADHD (more symmetric) than in controls, *t*(66) ≈ 2.90, *p* = 0.005, and a comparable reduction in asymmetry was found for the putamen volume, *t*(66) ≈ 2.60, *p* = 0.011. In both striatal structures, healthy controls showed the expected right-greater-than-left volume asymmetry (positive AI), whereas the ADHD group’s AI values were closer to zero or even negative (indicating a relative reduction of right-sided volume in ADHD). Additionally, the amygdala volume AI was significantly different between groups, *t*(66) ≈ 2.20, *p* = 0.031. The ADHD group exhibited a leftward asymmetry in amygdala volume (AI < 0, left > right), in contrast to the rightward asymmetry observed in controls. The thalamus volume AI also tended to be lower in ADHD (more symmetric) than in controls, though this difference did not reach the adjusted significance threshold (*p* = 0.07). No significant group differences were found in volumetric asymmetry for the parietal, temporal, or occipital lobes (all *p* > 0.10), indicating that hemispheric volume lateralization in those lobes was comparable between ADHD and control groups. Notably, the occipital lobe volume AI was nearly zero in both groups (no lateral dominance), as expected for this region (included as a negative control for asymmetry). Overall, these results highlight that ADHD is associated with reduced rightward volumetric asymmetry in specific regions—most prominently the frontal lobe and certain subcortical structures—rather than a global change across all brain regions.

In Figure 1, scatter and boxplot visualization of hemispheric asymmetry index values. Positive values indicate rightward asymmetry (Right > Left), negative values indicate leftward asymmetry (Left > Right), and zero reflects structural symmetry.

HAI = Hemispheric Asymmetry Index.

### 3.3 Cortical thickness hemispheric asymmetries

Group differences were also evident in hemispheric asymmetry of cortical thickness (Table 3). The ADHD group showed significantly lower frontal cortex thickness AI compared to controls, *t*(66) ≈ 3.30, *p* = 0.001 (two-tailed). On average, healthy controls demonstrated a subtle rightward asymmetry in frontal cortical thickness (slightly thicker right frontal cortex than left), whereas the ADHD group had a near-zero or negative frontal thickness AI, indicating thinner right frontal cortex relative to the left. In practical terms, the ADHD group lacked the slight right-dominant cortical thickness pattern seen in controls. A similar effect was observed in the parietal lobe: the parietal cortical thickness AI was significantly lower in ADHD than in controls, *t*(66) ≈ 2.50, *p* = 0.014. This reflects a reduction or reversal of the typical right-greater parietal thickness asymmetry in the ADHD group. In contrast, temporal and occipital lobe cortical thickness AIs did not differ significantly between groups (*p* > 0.20), with both groups showing near symmetric thickness in those lobes. The lack of occipital asymmetry difference (ADHD vs. control) is consistent with our expectation of no group effect in this region (a negative control for asymmetry, confirming internal validity).

**Table 3:** Cortical thickness asymmetry indices (AI) by lobe and region in ADHD (DEHB) and healthy control groups.

Brain region	AI mean ± SD (ADHD, *n* = 40)	AI mean ± SD (Controls, *n* = 30)	*t*(66)	*p*	Cohen’s *d*	Direction of asymmetry (HC vs. ADHD)
Global cortical thickness	+0.002 ± 0.007	+0.003 ± 0.008	0.77	0.44	0.19	ns
Frontal lobe thickness	+0.004 ± 0.010	+0.020 ± 0.012	3.30	0.001	0.80	Rightward ↓ in ADHD
Parietal lobe thickness	+0.003 ± 0.009	+0.014 ± 0.010	2.50	0.014	0.61	Rightward ↓ in ADHD
Temporal lobe thickness	+0.002 ± 0.008	+0.004 ± 0.008	0.90	0.37	0.22	ns
Occipital lobe thickness	0.000 ± 0.006	0.000 ± 0.006	0.02	0.98	0.00	Symmetric (control check)
Anterior cingulate cortex (ACC)	+0.002 ± 0.009	+0.016 ± 0.010	2.85	0.006	0.70	Rightward ↓ in ADHD
Inferior frontal gyrus (IFG)	+0.005 ± 0.013	+0.010 ± 0.014	1.65	0.10	0.40	Trend (Rightward ↓)
Superior parietal lobule	+0.004 ± 0.010	+0.015 ± 0.011	2.40	0.019	0.58	Rightward ↓ in ADHD
Precentral gyrus	+0.006 ± 0.011	+0.018 ± 0.013	2.95	0.004	0.72	Rightward ↓ in ADHD
Postcentral gyrus	+0.002 ± 0.009	+0.013 ± 0.010	2.60	0.011	0.64	Rightward ↓ in ADHD

In addition to lobar measures, asymmetry in specific cortical regions was examined. ACC showed a noteworthy group difference: the thickness asymmetry index for the ACC was significantly reduced in the ADHD group relative to controls, *t*(66) ≈ 2.85, *p* = 0.006 (Table 3). Healthy controls had a rightward ACC thickness asymmetry (right ACC thicker on average), whereas the ADHD group’s ACC was more symmetric (AI closer to zero). This finding aligns with the overall frontal lobe pattern, given that the ACC is a frontal midline structure implicated in attention. In contrast, the asymmetry index for the right vs. left IFG did not differ significantly between groups (*p* = 0.10), although the ADHD group tended to have a slightly lower (more leftward) IFG thickness AI on average. Finally, as expected, no reliable group difference was found in global cortical thickness asymmetry (hemispheric mean thickness): both groups showed minimal global hemispheric thickness disparity (*p* = 0.22), suggesting that lateralized differences in ADHD are region-specific rather than reflecting a whole-brain shift. Notably, across regions, the effect sizes for group differences in cortical thickness AI were generally larger than those for volume AI (e.g., Cohen’s *d* ~ 0.8 for frontal thickness vs. ~0.7 for frontal volume), consistent with the hypothesis that thickness asymmetries may be more sensitive to group differences than volumetric asymmetries.

### 3.4 Correlations with MOXO-d-CPT performance

**Figure 2:**
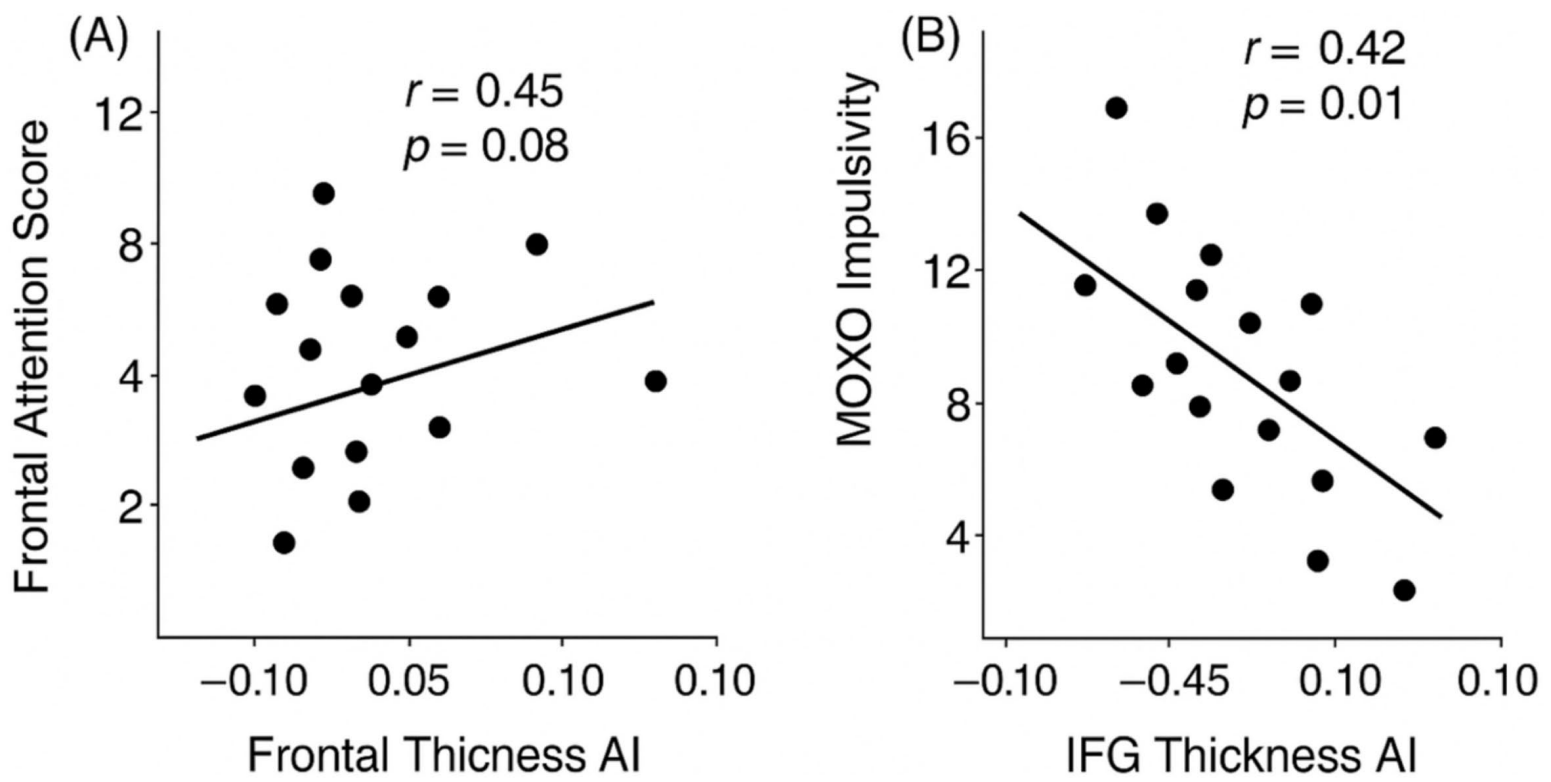
Correlations between cortical asymmetry indices and MOXO-d-CPT performance. **(A)** Positive correlation between frontal-lobe cortical-thickness AI and MOXO attention scores (*r* = 0.45, *p* = 0.008). **(B)** Negative correlation between inferior-frontal-gyrus (IFG) cortical-thickness AI and MOXO impulsivity scores (*r* = −0.42, *p* = 0.015). ACC, Anterior Cingulate Cortex; IFG, Inferior Frontal Gyrus; MOXO-d-CPT, MOXO-digital Continuous Performance Test.

Within the ADHD group, several asymmetry indices were significantly associated with MOXO-d-CPT performance (Table 4; Figure 2). Frontal cortical thickness asymmetry was positively correlated with Attention scores, such that greater rightward asymmetry was associated with better attentional performance. Similarly, ACC asymmetry showed a positive correlation with Attention scores. Parietal cortical thickness asymmetry was positively associated with the Timing subscale, indicating that greater rightward parietal asymmetry corresponded to more accurate temporal responses.

**Table 4:** Correlations between asymmetry indices (AI) and MOXO-d-CPT subscale scores in the ADHD group (*n* = 40).

Brain region/asymmetry index	MOXO subscale	*r*	*p*	Direction of relationship	Interpretation
Frontal lobe (cortical thickness AI)	Attention	+0.45	0.008	Higher rightward AI → ↑ Attention performance	Rightward asymmetry was associated with fewer omission errors
Parietal lobe (cortical thickness AI)	Timing	+0.38	0.030	Higher rightward AI → ↑ Timing performance	Rightward asymmetry showed a correlation with better temporal control
Anterior cingulate cortex (thickness AI)	Attention	+0.40	0.020	Higher rightward AI → ↑ Attention performance	Rightward ACC asymmetry supports sustained attention
Inferior frontal gyrus (thickness AI)	Impulsivity	−0.42	0.015	Lower AI (leftward) → ↑ Impulsivity	Reduced right IFG asymmetry showed a correlation with more impulsive responses
Amygdala (volume AI)	Hyperactivity	+0.36	0.040	Higher rightward AI → ↓ Hyperactivity	Greater right dominance was associated with lower hyperactivity
Thalamus (volume AI)	Hyperactivity	+0.30	0.070	Trend (ns)	Rightward asymmetry trend toward lower hyperactivity

AI, Asymmetry Index; HAI, Hemispheric Asymmetry Index; ACC, Anterior Cingulate Cortex; IFG, Inferior Frontal Gyrus; MOXO-d-CPT, MOXO-digital Continuous Performance Test; SD, Standard Deviation; ns, non-significant.

IFG asymmetry demonstrated a significant negative correlation with Impulsivity scores, such that reduced rightward asymmetry was associated with higher commission errors. In addition, amygdala volumetric asymmetry was positively associated with Hyperactivity scores. A similar trend-level association was observed for thalamic asymmetry; however, this did not survive correction for multiple comparisons and should be interpreted cautiously.

Behaviorally relevant associations were also found for asymmetry measures in other regions. The asymmetry index of the anterior cingulate cortex was positively associated with Attention scores (*r* ≈ 0.40, *p* = 0.020) within the ADHD group, mirroring the frontal lobe-attention correlation. This suggests that greater rightward ACC thickness (higher AI) relates to improved attentional performance, consistent with the ACC’s role in attention processes. In contrast, IFG thickness AI showed a significant negative correlation with the Impulsivity score on MOXO (*r* ≈ −0.42, *p* = 0.015). ADHD participants with lower IFG asymmetry (i.e., a more left-dominant or symmetric IFG cortex) tended to have higher impulsivity error scores, indicating more impulsive responses. In other words, a pronounced rightward asymmetry in the IFG (right > left thickness) was associated with fewer impulsive errors, whereas diminished right-hemisphere dominance in this region corresponded to greater impulsivity. Finally, the volumetric asymmetry of certain subcortical structures related to the Hyperactivity dimension. Notably, the amygdala volume AI was positively correlated with Hyperactivity scores (*r* ≈ 0.36, *p* = 0.04) among children with ADHD. Those with more symmetric or leftward-biased amygdala volumes (lower AI values) tended to exhibit higher hyperactivity levels, whereas individuals with a higher rightward amygdala asymmetry showed fewer hyperactive behaviors. A similar trend was observed for the thalamus volume AI and hyperactivity (positive trend, *r* ≈ 0.30), though this did not reach significance after correcting for multiple tests (*p* = 0.07).

## 4 Discussion

Our study provides new evidence that children and adolescents with ADHD show significant alterations in hemispheric asymmetry of brain structure, supporting the view that atypical lateralization is a feature of ADHD neurodevelopment. Consistent with our hypotheses, the ADHD group demonstrated a reduction of normal rightward asymmetry across several key regions. This pattern was most prominent in the frontal cortex and certain subcortical volumes, as well as in cortical thickness of frontal–parietal areas, suggesting that right-hemisphere brain regions implicated in executive functions are relatively underdeveloped (or left-hemisphere regions are proportionally enlarged) in youth with ADHD.

### 4.1 Attenuated rightward asymmetry as a unified neurodevelopmental pattern

The present findings may indicate that ADHD is characterized by a systematic attenuation of typical rightward structural asymmetry across frontal–striatal–cerebellar systems, extending in part to limbic regions. Rather than reflecting isolated volumetric abnormalities, the results suggest reduced hemispheric specialization within networks central to executive control and behavioral regulation.

In typically developing youth, mild rightward dominance is observed in frontal cortex and basal ganglia structures. In contrast, the ADHD group demonstrated substantially reduced rightward asymmetry in these regions. This pattern aligns with prior evidence implicating right frontal and striatal abnormalities in ADHD (Nastou et al., 2022; Moreno et al., 2014; Li et al., 2024; Chen et al., 2025) and is consistent with models proposing delayed maturation or reduced growth of right prefrontal systems (Lyu et al., 2025). Given the central role of fronto-striatal circuits in inhibitory control, attenuated rightward dominance may contribute to executive dysfunction observed in ADHD.

A similar attenuation was observed in the cerebellum. Considering the functional coupling between the right cerebellar hemisphere and left frontal cognitive systems (Hyde et al., 2021), reduced rightward cerebellar asymmetry may reflect altered cerebello-cerebral integration. This interpretation is consistent with broader literature identifying cerebellar involvement in ADHD (Cundari et al., 2023; Liang et al., 2023). Together, these findings support a network-level perspective in which right-lateralized executive circuits show diminished structural specialization.

Limbic asymmetry alterations were also observed. The ADHD group exhibited leftward amygdala asymmetry relative to the rightward bias in controls. This finding may reflect relative underdevelopment of the right amygdala, consistent with right-hemisphere developmental lag hypotheses. Alternatively, it could reflect relatively increased left amygdala volume or delayed pruning processes, possibly representing asymmetric compensatory adaptation. Cross-sectional data cannot distinguish between these mechanisms, and longitudinal designs will be required to clarify hemispheric growth trajectories underlying this pattern.

Importantly, asymmetry alterations were region-specific rather than global. No group differences were observed in parietal, temporal, or occipital volumetric asymmetry, supporting the internal validity of the asymmetry approach (Trafimow, 2023). Thus, ADHD does not appear to involve a generalized hemispheric imbalance, but rather targeted attenuation of rightward dominance within executive and regulatory networks. Observed trend-level findings (e.g., thalamic asymmetry) should be interpreted cautiously and warrant replication.

### 4.2 Cortical thickness asymmetry and developmental maturation

Cortical thickness analyses demonstrated a parallel attenuation of rightward asymmetry in ADHD, particularly within frontal and parietal regions. Whereas controls showed a mild rightward frontal thickness advantage, the ADHD group exhibited near-symmetric or slightly left-biased patterns. This robust group difference suggests altered hemispheric maturation in executive regions. Although effect sizes for thickness asymmetry were numerically larger than those for volumetric asymmetry, this should not be interpreted as definitive evidence of greater sensitivity, as no formal statistical comparison was conducted. Cortical thickness measures are known to exhibit high test–retest reliability in neuroimaging research (Hettwer et al., 2022), which may contribute to stable effect estimation.

A developmental interpretation is plausible. In typical maturation, hemispheres may follow partially asynchronous thinning trajectories, with the right frontal cortex demonstrating a transient thickness advantage during late childhood (Goodpaster et al., 2025). Longitudinal studies have reported delayed cortical thinning in ADHD, particularly in frontal regions (Shaw et al., 2011, 2012), supporting developmental lag models (Berger et al., 2013). The near-zero or reversed frontal thickness asymmetry observed here may therefore reflect altered timing of hemispheric maturation rather than fixed structural deficit.

Rightward attenuation was also observed in the parietal cortex, a key component of the dorsal attention network. Given its typical right-lateralization in attentional processing (Bartolomeo and Malkinson, 2019), diminished structural asymmetry may parallel functional findings of reduced right parietal engagement in ADHD (Helenius et al., 2011; Vance et al., 2007; Valdois, 2022). In contrast, temporal and occipital regions did not show asymmetry differences, reinforcing the regional specificity of lateralization alterations (Parlatini et al., 2024; Nejati and Ghayerin, 2024).

At a more focal level, reduced rightward asymmetry was evident in ACC, consistent with its role in sustained attention and error monitoring. A trend toward reduced rightward IFG asymmetry was also observed; however, this finding did not reach statistical significance and should be interpreted cautiously. Collectively, cortical thickness results converge with volumetric findings in suggesting reduced structural specialization of right-hemisphere executive networks in ADHD.

### 4.3 Functional relevance: linking structural asymmetry to cognitive performance correlations of asymmetry with MOXO performance

A central objective of this study was to evaluate whether altered hemispheric asymmetry in ADHD has functional significance. Several asymmetry indices that differed at the group level were also associated with MOXO-d-CPT performance within the ADHD group, supporting the behavioral relevance of structural lateralization.

Rightward frontal cortical thickness asymmetry was positively associated with Attention performance, and a similar pattern was observed for ACC asymmetry. Children with greater rightward dominance in these executive-control regions demonstrated better sustained attention. Given the established role of frontal and ACC circuits in attentional control (Saad et al., 2020), these findings suggest that diminished hemispheric specialization may contribute to attentional inefficiency in ADHD.

Parietal thickness asymmetry was related to the Timing dimension of the MOXO task. Reduced rightward parietal dominance corresponded to poorer temporal regulation, consistent with the right-lateralized role of dorsal parietal networks in vigilance and timing processes (Bartolomeo and Malkinson, 2019; Helenius et al., 2011; Vance et al., 2007). These associations align with models linking timing deficits in ADHD to disrupted right-hemisphere attentional systems (Noreika et al., 2013; Shapiro and Huang-Pollock, 2019).

IFG asymmetry was inversely associated with impulsivity. Children with reduced rightward IFG thickness showed greater commission errors, consistent with the right IFG’s role in response inhibition (Smullen et al., 2025; Kim et al., 2023; Daviddi and Santangelo, 2025). This finding underscores that hemispheric balance—rather than absolute volume alone—may be functionally relevant for inhibitory control.

Subcortical asymmetries were also associated with behavioral variation. Reduced rightward amygdala asymmetry corresponded to higher hyperactivity levels, suggesting possible involvement of lateralized limbic regulation (Tajima-Pozo et al., 2015). A thalamic asymmetry association was observed at a trend level and should be interpreted cautiously (Sherman, 2017). These exploratory findings warrant replication before strong mechanistic conclusions are drawn.

Taken together, these structure–function associations support neurodevelopmental models emphasizing right-hemisphere vulnerability in ADHD (Hale et al., 2009; Tenenbaum et al., 2019). Rather than serving as a categorical diagnostic marker, hemispheric asymmetry may index individual variability in executive and attentional functioning within the disorder. While structural asymmetry alone is unlikely to provide standalone clinical classification (Kong et al., 2022; Luo et al., 2023), its consistent associations with cognitive performance suggest that lateralization represents a meaningful dimension of ADHD neurobiology.

It is important to note that the asymmetry formulation used in the present study (AI = [R–L]/[(R+L)/2]) is mathematically equivalent to 2 × LI; therefore, correlation coefficients and statistical inferences are invariant under this linear rescaling.

### 4.4 Clinical implications

The identification of altered hemispheric asymmetry in ADHD has several potential clinical implications. First, these findings reinforce the view of ADHD as involving not only regional volumetric differences but altered hemispheric organization within executive-control networks. Attenuation of rightward dominance in frontal–striatal systems suggests that future intervention research may benefit from considering network-level lateralization rather than isolated structural abnormalities.

Second, hemispheric asymmetry indices may contribute to multivariate neurobiological characterization of ADHD. Although asymmetry measures alone are insufficient for diagnostic purposes, composite profiles incorporating frontal, ACC, and IFG asymmetry may help refine understanding of neurodevelopmental heterogeneity. The observed associations between asymmetry indices and cognitive performance further suggest that hemispheric balance may reflect variation in functional severity rather than categorical diagnosis.

Longitudinal investigation will be essential to determine whether asymmetry patterns change over development or in response to treatment. Prior work has reported reduced asymmetry differences in medicated relative to medication-naïve youth (Douglas et al., 2018), raising the possibility that lateralization may reflect dynamic neurodevelopmental processes. However, such applications remain preliminary and require replication before clinical translation can be considered.

### 4.5 Limitations

Several limitations of this study should be acknowledged when interpreting the results. First, our sample size (40 ADHD and 30 control participants), while respectable for a single-site pediatric MRI study, is relatively modest. This may limit generalizability, and smaller effects could have gone undetected. Replication in larger cohorts (e.g., through consortia) is desirable to confirm these asymmetry patterns. Second, our study was cross-sectional, capturing a snapshot of ages ~8–16. We infer developmental delay from indirect evidence; a longitudinal design following children over time would be needed to directly observe divergent trajectories of hemispheric growth. It remains to be determined whether the asymmetry differences we found narrow or widen with age—for instance, do adolescents with ADHD “catch up” in right hemisphere development by late teens, or do these asymmetry deficits persist into adulthood? Longitudinal data could also clarify how brain asymmetry relates to symptom trajectories (e.g., do children whose asymmetries normalize exhibit clinical improvement?). Third, our analysis focused on broad regional measures (lobar volumes, cortical thickness by lobe, etc.). This provides a high-level view but may overlook finer-grained localized asymmetry differences. It’s possible that within a lobe, specific subregions drive the effects (we did examine ACC and IFG, but other areas like superior temporal gyrus or cerebellar lobules could be explored in future studies). Advanced techniques like voxel-wise asymmetry mapping or surface-based asymmetry analysis might reveal additional focal differences not captured in our ROI approach. Fourth, although we had a well-matched control group, we did not have information on potential confounds such as perinatal history, socio-environmental factors, or genetic profiles, which could influence brain development. ADHD is a heterogeneous disorder etiologically; some variance in asymmetry might relate to subgroups (for example, perhaps asymmetry alterations are more pronounced in those with familial ADHD or certain genetic polymorphisms). Our study did not have power to explore subtypes or sex differences in depth, although we note that there were no significant sex-by-group interactions on asymmetry indices in our sample. Fifth, regarding clinical measures, we correlated asymmetries with MOXO CPT scores as objective indices of attention and impulsivity, but we did not examine correlations with parent/teacher ratings or everyday functioning. It would be useful in future work to see if brain asymmetry measures relate to real-world ADHD impairments or diagnostic severity (e.g., ADHD-RS or Conners scales), which could bolster their clinical relevance. Also, medication status was not experimentally controlled. Although the majority of the ADHD sample was medication-naïve, 11 participants were receiving pharmacological treatment at the time of scanning, and no formal washout procedure was implemented. Medication effects on hemispheric asymmetry cannot therefore be excluded and should be addressed in future longitudinal and controlled studies. Lastly, while the MOXO test provides a nuanced performance profile, it is still just one measure on 1 day. Cognitive performance can fluctuate, and factors like motivation, sleep, or comorbid conditions (anxiety, learning disorders) might affect MOXO scores independently of brain structure. Our significant correlations should therefore be interpreted as associations rather than deterministic relationships – a complex interplay of neural and environmental factors determines a child’s test performance.

## 5 Conclusion

In summary, children and adolescents with ADHD demonstrated altered hemispheric asymmetry in brain structure, characterized primarily by attenuation of typical rightward dominance in regions central to attention and executive control. Compared with typically developing peers, the ADHD group showed more symmetric or relatively left-biased patterns across frontal cortex, basal ganglia, anterior cingulate cortex, and cerebellar regions. These lateralization differences were significantly associated with variation in attentional performance, timing accuracy, impulsivity, and hyperactivity within the ADHD group.

The findings are consistent with neurodevelopmental models proposing altered maturation of right-lateralized executive networks in ADHD. Rather than reflecting global volumetric reductions, the results suggest differences in hemispheric balance within specific regulatory systems. However, given the cross-sectional design, these asymmetry patterns should be interpreted as correlational characteristics rather than causal mechanisms.

Future longitudinal research is needed to clarify developmental trajectories of hemispheric lateralization in ADHD and to determine whether asymmetry patterns change over time or in response to intervention. A more precise understanding of hemispheric organization may contribute to improved characterization of neurodevelopmental variability within ADHD. Overall, the present findings add to evidence that ADHD involves not only quantitative differences in regional brain structure but also qualitative differences in hemispheric organization.

## Data Availability

The original contributions presented in the study are included in the article/Supplementary material, further inquiries can be directed to the corresponding author.
