# Supplementary material for: Attenuated rightward hemispheric asymmetry in ADHD: structural MRI evidence from a normalized asymmetry index and its association with cognitive performance
Source: Front Neurosci. 2026 Mar 12;20:1764242. doi: 10.3389/fnins.2026.1764242 (PMC13017821; doi:10.3389/fnins.2026.1764242)
Supplement: Supplementary file 1 [file Data_Sheet_1.DOCX]

**Supplementary Methods S1**

**MOXO-d-CPT Scoring Procedures**

The MOXO-d-CPT is a computerized continuous performance test designed to assess attentional control and response inhibition in children and adolescents. The test generates four standardized indices: Attention, Timing, Impulsivity, and Hyperactivity. Scores are age- and sex-normed.

**Attention Index:**
Primarily reflects omission errors and consistency of correct responses. Omission errors occur when a participant fails to respond to a target stimulus. Greater omission frequency and inconsistent response patterns result in lower Attention scores. Reaction time variability contributes to performance stability assessment (Berger et al., 2013).

**Timing Index:**
Represents the accuracy of response timing relative to target stimulus presentation. Responses are classified as correct only if they occur within a predefined temporal window. Early or delayed responses reduce the Timing score. This index captures temporal regulation and vigilance efficiency.

**Impulsivity Index:**
Based on commission errors, defined as responses to non-target stimuli. A higher number of commission errors reflects greater impulsive responding and results in lower standardized Impulsivity scores.

**Hyperactivity Index:**
Reflects excessive motor responses, including repetitive or unnecessary key presses beyond target requirements. This index captures motor overactivity independent of omission and commission errors.

Scoring procedures are derived from the MOXO-d-CPT technical manual and validation literature (Berger et al., 2013).
