# Supplementary material for: Attenuated rightward hemispheric asymmetry in ADHD: structural MRI evidence from a normalized asymmetry index and its association with cognitive performance
Source: Front Neurosci. 2026 Mar 12;20:1764242. doi: 10.3389/fnins.2026.1764242 (PMC13017821; doi:10.3389/fnins.2026.1764242)
Supplement: Supplementary file 2 [file Data_Sheet_2.DOCX]

**Supplementary Methods S2**

In the volBrain analysis pipeline, the “scale factor” refers to the global scaling coefficient applied during transformation of the uploaded T1-weighted magnetic resonance image into standard reference space. The volBrain system automatically performs several preprocessing steps, including bias field correction, skull stripping (removal of non-brain tissues), gray matter–white matter–cerebrospinal fluid segmentation, and affine normalization to standard space (typically the MNI template). The global expansion or contraction ratio applied during this affine normalization step is reported as the “scale factor.”

A scale factor value close to 1.00 indicates that the individual’s brain size is similar to the reference template. Values greater than 1.00 indicate that the image was scaled up to match the template (suggesting that the individual brain may be smaller than the template), whereas values less than 1.00 indicate that the image was scaled down (suggesting that the individual brain may be larger than the template). Importantly, this parameter does not directly represent biological atrophy or hypertrophy. Rather, it reflects a technical adjustment arising from individual differences in head and brain size during normalization to standard space.

Clinically, the scale factor alone is not a meaningful biomarker. Primary structural evaluations are based on intracranial volume (ICV), gray and white matter volumes, regional volumetric measurements, and age-adjusted normative Z-scores. The scale factor is primarily relevant for quality control and normalization procedures. In longitudinal studies in particular, substantial changes in scale factor values are more likely to reflect differences in imaging parameters, scanner variation, or preprocessing inconsistencies rather than true biological volume loss.

| *Independent Samples T-Test* | | | | | | | |
| --- | --- | --- | --- | --- | --- | --- | --- |
|  | | t | | df | | p | |
| Scale_factor |  | -0.012 |  | 63 |  | .991 |  |
|  | | | | | | | |
| *Note.*  Student's t-test. | | | | | | | |

**Descriptives**

| *Group Descriptives* | | | | | | | | | | | | | |
| --- | --- | --- | --- | --- | --- | --- | --- | --- | --- | --- | --- | --- | --- |
|  | | Group | | N | | Mean | | SD | | SE | | Coefficient of variation | |
| Scale_factor |  | ADHD (1) |  | 40 |  | 0.767 |  | 0.085 |  | 0.016 |  | 0.110 |  |
|  |  | HCs (0) |  | 30 |  | 0.768 |  | 0.081 |  | 0.013 |  | 0.106 |  |
|  | | | | | | | | | | | | | |


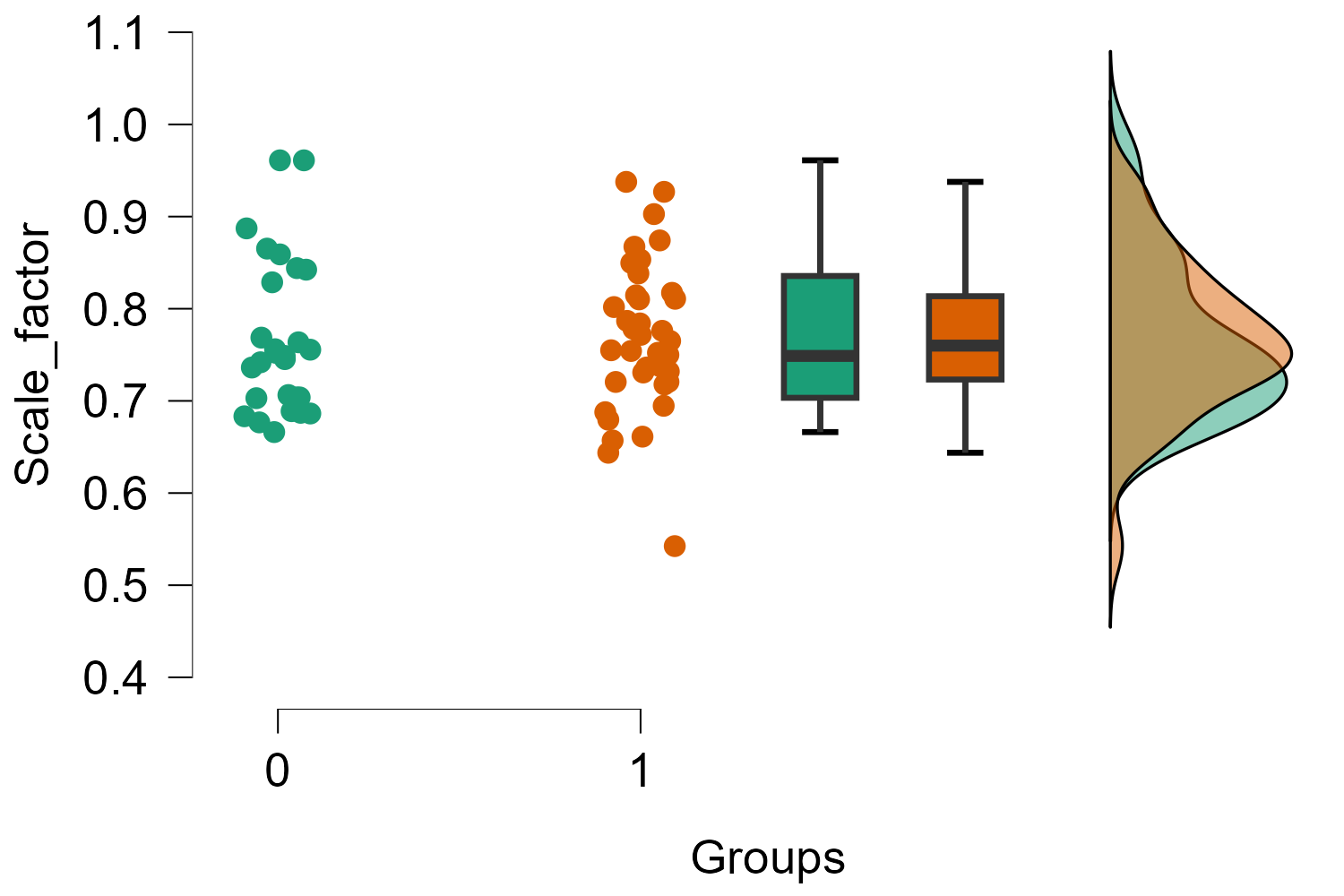


The scale factor in the volBrain analysis represents the global scaling ratio applied to the T1-weighted MRI image during normalization to the MNI template.

- A value approximately equal to 1.00 indicates that the individual’s brain size is similar to the reference template.
- Values greater than 1.00 indicate that the image was scaled up to match the template (suggesting the individual brain may be smaller than the template).
- Values less than 1.00 indicate that the image was scaled down (suggesting the individual brain may be larger than the template).

Importantly, the scale factor is a technical normalization parameter and does not reflect biological atrophy or hypertrophy.

The comparison of scale factor values between the ADHD and healthy control (HC) groups yielded a non-significant result (p = .991), indicating no statistically meaningful difference in global scaling between groups. Mean scale factor values were nearly identical (ADHD: ~0.767; HC: ~0.768), both clearly below 1.00. This suggests that, on average, brain size in both groups was larger than the MNI template, requiring downscaling during normalization. Standard deviations and coefficients of variation were also comparable between groups, and visual inspection (boxplot analysis) showed highly similar distributions. No outliers were observed in the ADHD group; a minor outlier may have been present in the control group, but it did not influence group-level results.

Overall, no difference in scale factor was observed between ADHD and HC groups. This is expected, as scale factor reflects technical normalization rather than biological variation. The absence of group differences supports the conclusion that MRI preprocessing and spatial normalization were comparable across groups. Consequently, any observed differences in gray matter volume, intracranial volume (ICV), or regional measures are unlikely to be attributable to normalization artifacts and are more plausibly interpreted as biological rather than technical effects.
